# TET1: The epigenetic architect of clinical disease progression

**DOI:** 10.1016/j.gendis.2025.101513

**Published:** 2025-01-04

**Authors:** Keyvan Jabbari, Ali Khalafizadeh, Mahboubeh Sheikhbahaei, Hossein Soltaninejad, Sadegh Babashah

**Affiliations:** a Department of Molecular Genetics, Faculty of Biological Sciences, Tarbiat Modares University, Tehran 14115-154, Iran; b Department of Stem Cells Technology and Tissue Regeneration, Faculty of Interdisciplinary Sciences and Technologies, Tarbiat Modares University, Tehran 14115-154, Iran

**Keywords:** Clinical diseases, DNA demethylation, Epigenetics, TET1, Therapeutic target

## Abstract

The ten-eleven translocation 1 (TET1) protein, a member of the human α-ketoglutarate-dependent dioxygenase TET family, functions as a 5-methylcytosine hydroxylase with a strong affinity for genomic regions enriched with 5′-CpG-3′ dinucleotides, particularly CpG islands. TET1 is critical in initiating DNA demethylation and maintaining a balanced interaction between demethylation and DNA methylation, which is essential for genomic methylation stability and precise epigenetic regulation. By removing methyl groups from specific tumor suppressor genes, TET1 can influence their expression. This review summarizes the latest advancements in TET1 research, emphasizing its role in demethylation mechanisms and its significance in regulatory processes related to clinical conditions. TET1 is a crucial mediator of demethylation, although the precise details of this mechanism are not yet fully understood. Additionally, TET1 plays a key role in inhibiting tumor progression, but its effects vary across different tumors. This variability arises from its interactions with diverse signaling pathways, where it can function either as an antagonist or a promoter. The role of TET1 remains controversial in certain cancer types, and its potential oncogenic functions have attracted growing interest, opening new avenues for investigation.

## Introduction

**Figure 1:**
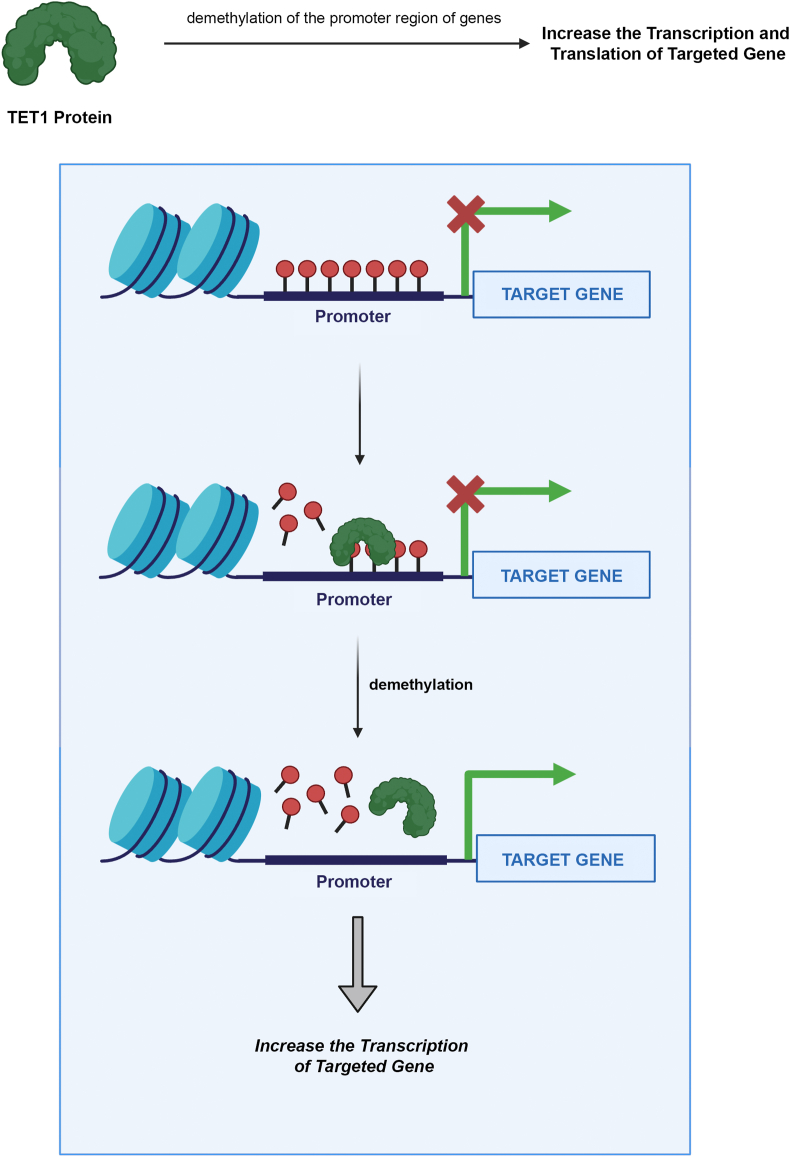
TET1-mediated transcriptional activation through promoter demethylation. This figure illustrates the mechanism by which TET1 facilitates gene transcription through the demethylation of promoter regions. The schematic diagram demonstrates TET1's enzymatic activity, which catalyzes DNA demethylation, ultimately leading to the activation of gene expression.

DNA methylation is a crucial epigenetic modification that significantly influences genome stability, transcriptional processes, and development. It involves adding a methyl group at the C5′ position of 5′-CpG-3′ dinucleotides, leading to the formation of 5-methylcytosine (5 mC). This chemical conversion is facilitated by DNA methyltransferases (DNMTs), which use S-adenosyl methionine as the active methyl donor. It is important to note that DNA methylation does not alter the actual sequence of DNA base pairs and has reversible biological properties.^1^^,^^2^ In other words, the transformation of 5 mC back to 5-cytosine can occur through the action of demethylase enzymes following DNA methylation. The balance of genomic methylation levels is maintained by the coordinated activities of DNMTs and demethylases within cells, a process essential for preserving stable DNA methylation patterns throughout the genome. Disruptions in this balance can lead to various diseases, including cancer.^3^, ^4^, ^5^, ^6^ The ten-eleven translocation (TET) enzymes form a group of methylcytosine dioxygenases known as TET enzymes.^7^^,^^8^ These enzymes, comprising TET1, TET2, and TET3, play a crucial role in epigenetic regulation by actively demethylating 5 mC to 5-hydroxymethylcytosine (5hmC), 5-formylcytosine, and 5-carboxylcytosine. This process influences gene expression patterns across various biological contexts (Fig. 1).^9^, ^10^, ^11^ Through base excision repair, 5-formylcytosine and 5-carboxylcytosine present in the DNA sequence are corrected, with these modified bases being replaced by cytosine.^12^ The primary shared function of TET proteins is their role in active DNA demethylation, which is essential for maintaining the epigenetic landscape and influencing cellular differentiation, gene expression, and disease progression.^13^ Despite these shared functions, TET proteins display distinct expression patterns and play specific roles in different cell types and developmental stages.^14^ TET1 is highly expressed in embryonic stem cells and primordial germ cells, TET2 is abundant in hematopoietic cells, and TET3 is predominantly expressed in oocytes and zygotes where it plays a crucial role in early embryonic development. Regarding genomic localization, TET1 preferentially binds to CpG-rich promoters, TET2 is primarily found at enhancers and gene bodies, and TET3 associates with both promoters and gene bodies, showing a preference for transcriptionally active regions.^15^^,^^16^ Developmentally, TET1 is essential for embryonic development and the maintenance of pluripotency, while TET2 plays a key role in hematopoietic differentiation. TET3, on the other hand, has a unique function in demethylating the paternal genome after fertilization, which is crucial for activating early embryonic genes.^17^^,^^18^ In cancer, TET1 has been associated with various solid tumors, including breast and bladder cancer (as discussed in this review), while TET2 is frequently mutated in hematological malignancies. Recent studies also suggest that TET3 may play a role in certain leukemias and solid tumors, such as glioblastoma and colorectal cancer.^19^ Beyond cancer, TET3 has specific implications in other clinical contexts. It regulates activity-dependent gene expression and memory formation in the brain, suggesting a role in neurodegenerative diseases and cognitive disorders. Additionally, TET3 is involved in metabolic regulation, including adipocyte differentiation and glucose homeostasis, indicating its potential role in metabolic diseases such as obesity and diabetes.^20^, ^21^, ^22^ The functions of TET1, TET2, and TET3 are often interconnected, with complementary or compensatory roles in various biological contexts. For example, in embryonic stem cells, TET1 and TET2 exhibit redundancy in maintaining pluripotency, while TET3 becomes critical during early development. In hematopoietic cells, TET2 is the predominant enzyme, but TET1 and TET3 can partially compensate for its loss. Additionally, in neuronal tissues, all three TET proteins are expressed and contribute to brain development and function, each playing overlapping yet distinct roles.^23^^,^^24^ Understanding the interplay between TET1, TET2, and TET3 is crucial for fully grasping TET-mediated epigenetic regulation and its clinical implications. While this review focuses primarily on TET1, acknowledging the roles of TET2 and TET3 offers a more comprehensive perspective on TET functions in both health and disease.

TET1, a key member of the TET family, has garnered significant attention in the context of clinical diseases due to its crucial role in epigenetic regulation and DNA demethylation. It is also important to recognize the various interactions that influence TET1's function in different biological contexts. One particularly notable interaction involves microRNAs (miRNAs). Recent studies have revealed a complex relationship between miRNAs and TET1 in regulating DNA methylation dynamics. These small, non-coding RNA molecules act as regulators of TET1 levels and activity by targeting its mRNA. Demethylation can activate previously silenced miRNAs, enhance transcription by improving transcription factor accessibility, and contribute to tissue-specific expression patterns. Altered miRNA expression, in turn, impacts key cellular processes such as proliferation, differentiation, and apoptosis. The regulatory relationship between miRNAs and TET1 plays a crucial role in the development of various clinical conditions, including cancer, highlighting its involvement in disease progression and potential therapeutic strategies. For example, TET1-mediated demethylation of tumor suppressor miRNA promoters can reactivate their expression, inhibiting tumor progression, while demethylation of oncogenic miRNA promoters may promote malignancy.^25^^,^^26^ Below are several areas in which TET1 has been implicated in clinical conditions.

## The role of TET1 in cancer progression and epigenetic regulation

TET1 has been implicated in various types of cancer due to its role in maintaining DNA methylation patterns and influencing gene expression, which are crucial for cancer progression and development. Dysregulation of TET1 function or alterations in its genetic structure can disrupt epigenetic regulation, potentially contributing to tumor growth, metastasis, and other cancer-related processes.

### Bladder cancer

The involvement of TET1 in bladder cancer progression is an area of growing interest. In 2018, Hu et al^27^ examined the interaction between TET1 and the long noncoding RNA XIST (X inactive specific transcript), demonstrating that TET1 contributes to the regulation of p53 expression through its interaction with XIST. This regulatory axis suggests that XIST-mediated repression of p53, facilitated by TET1, may play a critical role in bladder cancer progression. The mechanism involves XIST acting as a competing endogenous RNA (ceRNA) that sequesters microRNAs targeting TET1, thereby enhancing its expression. The elevated levels of TET1 then cause hypomethylation of the p53 promoter region, which paradoxically leads to reduced p53 expression, contrary to the usual correlation between promoter hypomethylation and increased gene expression. In 2020, Yan et al^28^ demonstrated that TET1 expression is reduced in urinary breast cancer samples compared with normal urothelium. This decrease is associated with tumor stage, grade, and overall survival, suggesting TET1's potential involvement in urinary breast cancer progression. Down-regulation of TET1 led to increased invasiveness and cell proliferation in urinary breast cancer cells while restoring normal TET1 expression counteracted these effects and suppressed tumor growth in animal models. The study revealed that reduced TET1 levels hindered hydroxymethylation of the AJAP1 (adherens junctions associated protein 1) promoter, thereby activating β-catenin signaling and promoting urinary breast cancer development. Another study by Mao et al^29^ suggested that OCT3/4 may promote tumor immune evasion in bladder cancer by enhancing the TET1–nuclear factor erythroid 2-related factor 2 (NRF2)–MDM2 pathway.

### Breast cancer

Breast cancer exhibits varying levels of DNA methylation, although the factors that regulate the balance between methylation and demethylation remain unclear. Notably, DNA demethylation in breast cancer is associated with metastasis, invasion, and cell proliferation. It has been shown that reducing TET1 expression enhances cell invasion, metastasis, and tumor growth in xenograft models, which correlates with poorer survival in breast cancer patients. TET1 regulates the expression of tissue inhibitors of metalloproteinase (TIMP) family proteins 2 and 3 (TIMP2 and TIMP3) by inhibiting DNA methylation. Low levels of TET1, TIMP2, and TIMP3 expression are commonly observed in samples with advanced nodal status, suggesting their involvement in disease progression.^30^ Subsequent studies have further highlighted TET1's role in breast cancer. Wielscher et al^31^ found decreased mRNA expression of both leucine zipper tumor suppressor 1 (LZTS1) and TET1 in tumor samples, with reductions in 5hmC levels linked to unfavorable histopathological features, including lymph node involvement. This supports the potential of 5hmC as a breast cancer biomarker and underscores its role in cancer progression. Sun et al^32^ discovered the high mobility group AT-hook 2 (HMGA2)–TET1–homeobox A9 (HOXA9) pathway, establishing it as a prognostic marker for breast cancer survival. In 2015, Sang et al^33^ observed that TET1 down-regulation via siRNA inhibited breast cancer cell invasion and was associated with lymph node metastasis.

**Figure 2:**
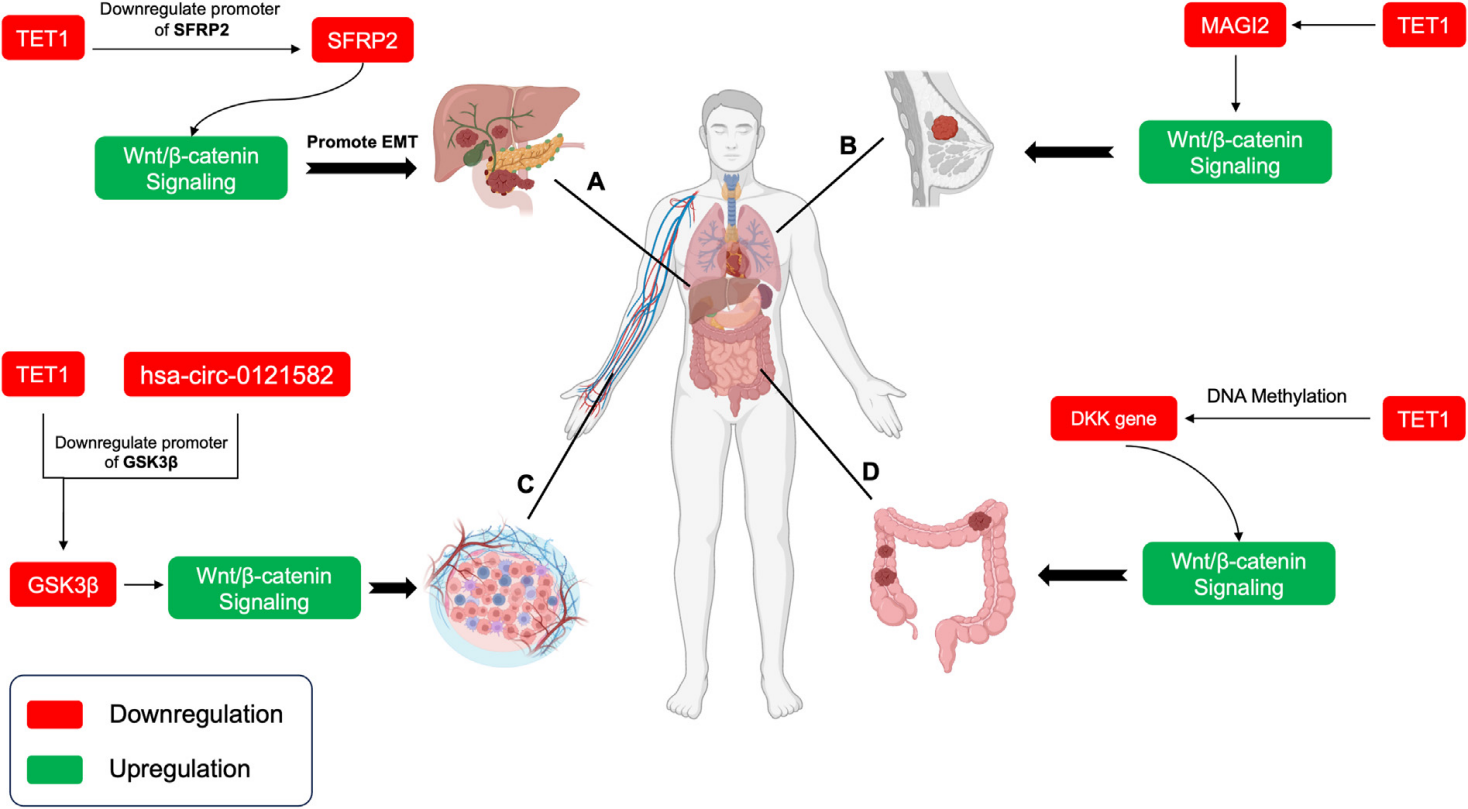
TET1 regulation of the Wnt/β-catenin pathway in various cancer types. This figure illustrates several instances of TET1's role in regulating the Wnt/β-catenin pathway across different cancer types. **(A)** TET1 promotes pancreatic tumor progression and metastasis by enhancing Wnt signaling. It interacts with the SFRP2 promoter, catalyzing demethylation to increase transcription, thereby activating both canonical and noncanonical Wnt pathways. **(B)** In breast cancer, a TET1 inhibitor reverses MAGI2-mediated Wnt suppression, restoring cell proliferation and migration. **(C)** In leukemia, hsa_circ_0121582 recruits TET1 to enhance Wnt signaling, leading to increased β-catenin accumulation and enhanced leukemia cell proliferation. **(D)** In colon cancer, TET1 promotes the demethylation of DKK gene promoters, activating DKK gene expression. Reduced TET1 levels in colon cancer lead to DKK gene hypermethylation, contributing to Wnt/β-catenin pathway activation, tumor invasion, and proliferation.

Research has also explored TET1's broader role in oncogenesis. In 2018, Good et al^34^ showed that TET1 contributes to hypomethylation and activates oncogenic factors such as phosphoinositide 3-kinase (PI3K), epidermal growth factor receptor (EGFR), and platelet-derived growth factor (PDGF), with TET1 expression correlating with sensitivity to PI3K–mechanistic target of rapamycin (mTOR) pathway inhibitors. After TET1 deletion in triple-negative breast cancer cell models, decreased expression of PI3K pathway genes, up-regulation of immune response genes, and reduced cell proliferation were observed. Additionally, Collignon et al^35^ uncovered a novel immune-related mechanism for TET1 regulation in basal-like breast cancer. Their findings suggested that nuclear factor-kappa B (NF-kB) activation could suppress TET1 via p65 binding, with low TET1 expression correlating with elevated immune markers in basal-like breast cancer tissues. Further insights into TET1's regulatory roles have emerged in recent years. In 2019, Yu et al^36^ identified an EZH2 (enhancer of zeste homolog 2)-tri-methylation of histone 3 at lysine 27 (H3K27me3)-TET1 module, showing that EZH2 suppression of TET1 impedes the anti-tumor p53 pathway, impacting survival rates in triple-negative breast cancer patients. Targeting EZH2-induced cell cycle arrest and senescence by enhancing TET1 and activating the p53 pathway. In 2020, Xu et al^37^ demonstrated that a TET1 inhibitor could reverse the effects of MAGI2-AS3 (MAGI2 antisense RNA 3) overexpression on the Wnt–β-catenin pathway, restoring cell proliferation and migration (Fig. 2). In 2022, Umeh-Garcia et al^38^ utilized CRISPR/dCas9 to demonstrate that TET1-mediated demethylation, combined with transcriptional activation of leucine-rich repeat and immunoglobulin-like domain 1 (LRIG1), could reduce breast cancer cell viability, offering a promising avenue for targeted breast cancer therapies.

### Blood cancer

The discovery of the TET gene family began with TET1 being identified as a fusion partner of mixed-lineage leukemia (MLL) in acute myeloid leukemia,^39^ a translocation also observed in T-cell lymphoma^40^ and B-cell acute lymphoblastic leukemia.^41^ The tumor-suppressive role of TET1 in hematopoietic malignancies was highlighted by Cimmino et al,^42^ who demonstrated that TET1 deletion in mice induced B-cell lymphoma with mutations resembling those found in non-Hodgkin B-cell lymphoma, accompanied by hypermethylation and silencing of TET1. In 2016, Jiang et al^43^ demonstrated that in acute myeloid leukemia, miR-22 was suppressed through epigenetic repression involving TET1, along with SIN3 transcription regulator family member A (SIN3A), EZH2, and growth factor independent 1 (GFI1), as well as DNA copy-number loss. This suppression highlighted the critical role of miR-22 in regulating the CREB and MYC pathways by targeting oncogenes such as CREB regulated transcription coactivator 1 (CRTC1), Fms-related receptor tyrosine kinase 3 (FLT3), and MYC binding protein (MYCBP). They later identified compounds inhibiting TET1 and 5hmC modifications, effectively reducing cell viability in acute myeloid leukemia with high TET1 levels, including t(11q23)/MLL rearrangements and t(8; 21) acute myeloid leukemia.^44^ Concurrently, Jamalpour et al^45^ reported a network involving SH2 domain-containing adaptor protein B (SHB), B-cell lymphoma 6 corepressor like 1 (BCORL1), and TET1 that enhances lymphoid traits, vascular development, and leukemia cell survival. Additional findings underscored the therapeutic potential of TET1 modulation. Shenoy et al^46^ demonstrated that ascorbic acid enhances SMAD1 expression through TET-mediated demethylation, thereby sensitizing lymphoma cells to chemotherapy. Moreover, Kim et al^47^ revealed that the MLL-TET1 fusion protein inhibits myeloid differentiation by up-regulating tribbles pseudokinase 2 (Trib2), a crucial factor for leukemia cell survival and differentiation arrest.

High TET1 expression has been associated with poor outcomes in acute myeloid leukemia. Wang et al^48^ linked TET1 expression to M0/M1 morphology and nucleophosmin 1 (NPM1) mutations, correlating with worse survival rates, particularly in acute myeloid leukemia without CCAAT enhancer binding protein alpha (CEBPA) mutations. Poole et al^49^ demonstrated the contrasting roles of TET1 and TET2 in T-cell acute lymphoblastic leukemia. TET1 promotes tumor growth by regulating genes involved in ribosomal biogenesis, while TET2 suppresses tumorigenesis. Inhibiting TET1 or activating TET2 was found to reverse tumor progression. In 2020, Bamezai et al^50^ showed that PARP enzymes enhanced TET1 expression in T-cell acute lymphoblastic leukemia by creating tri-methylation of histone 3 at lysine 4 (H3K4me3) marks on its promoter, and the poly(ADP-ribose) polymerase (PARP) inhibitor olaparib reduced TET1 expression, 5hmC levels, and tumor growth. Further studies in acute myeloid leukemia have provided additional insights. Zhang et al^51^ found a significant reduction in TET1 expression alongside elevated TET2 and TET3 levels, suggesting a complex role of TET enzymes in acute myeloid leukemia pathogenesis. In parallel, Chen et al^52^ discovered that circular RNA circ_0121582 could bind to the glycogen synthase kinase 3 beta (GSK3β) promoter in the nucleus, recruiting TET1 to facilitate GSK3β transcription. This increase in GSK3β inhibits the Wnt–β-catenin signaling pathway, reducing β-catenin accumulation in the nucleus and limiting leukemia cell proliferation (Fig. 2).

TET1 is also a promising target for therapeutic interventions. Homoharringtonine, approved for chronic myeloid leukemia, inhibits acute myeloid leukemia growth by targeting the specificity protein 1 (SP1)/TET1 axis and reducing 5hmC levels. This epigenetic mechanism, combined with FLT3 signaling inhibition, underscores its potential in treating FLT3-mutated acute myeloid leukemia.^53^ Recently, Chen et al^54^ uncovered a carcinogenic role for phosphorylated TET1 in B-cell acute lymphoblastic leukemia, driven by protein kinase C epsilon (PRKCE) and ataxia-telangiectasia mutated (ATM) kinases. Overexpression of TET1 transformed normal B cells into B-cell acute lymphoblastic leukemia cells by promoting the transcription of cluster of differentiation 72 (CD72) and joining chain of multimeric IgA and IgM (JCHAIN) through signal transducer and activator of transcription 5B (STAT5B) interactions. Targeting TET1 stability or its associated pathways significantly reduced B-cell acute lymphoblastic leukemia cell viability, presenting new therapeutic avenues, particularly for refractory or relapsed cases. Collectively, these findings highlight TET1's dual role in both promoting tumorigenesis and serving as a potential therapeutic target across various hematologic malignancies.

### Lung cancer

The role of TET1 in lung cancer continues to be an area of active research and debate. In 2016, Taylor et al^55^ highlighted TET1's involvement in squamous cell carcinoma, where its regulation is mediated through a mitogen-activated protein kinase (MAPK)-driven pathway. In the immune-evasion subtype, the MAPK-driven E26 transformation-specific 1 (ETS1) transcription factor regulates miR-29b, which down-regulates TET1 and leads to reduced 5hmC modifications. Inhibiting MAPK signaling restored TET1 expression and increased 5hmC levels, emphasizing the influence of MAPK signaling on TET1 regulation. Forloni et al^56^ investigated this topic, demonstrating that oncogenic EGFR silenced tumor suppressor genes in lung adenocarcinomas and glioblastomas by inhibiting TET1 through C/EBPα. Inhibition of EGFR allowed TET1 to bind to tumor suppressor promoters, reactivating their expression via DNA demethylation and significantly inhibiting tumor growth. Conversely, TET1 knockdown led to resistance to EGFR inhibitors. In most lung cancer cases, TET1 expression is either reduced or predominantly localized in the cytoplasm, limiting its tumor-suppressive potential. The functional importance of TET1 in lung cancer has been further emphasized in studies exploring its interactions and regulatory networks. Yokoyama et al^57^ demonstrated that TET1-driven DNA hypomethylation regulated mucin 4 (MUC4) expression in lung cancer, with reduced TET1 levels leading to decreased MUC4 levels. In lung adenocarcinoma, Pei et al^58^ discovered that TET1 formed a complex with methyl-CpG binding domain protein 2 (MBD2) to activate miR-200s, key regulators of tumor invasiveness. Reduced MBD2 levels were associated with increased metastasis and poorer survival in lung adenocarcinoma patients. Additionally, Filipczak et al^59^ found that in p53-mutated lung cancers, TET1 expression was elevated, and its suppression induced senescence marked by genomic instability, DNA damage, and increased p21 expression. Combining TET1 inhibition with chemotherapy enhanced this effect, highlighting its dual role in both tumor progression and therapeutic response.

**Figure 3:**
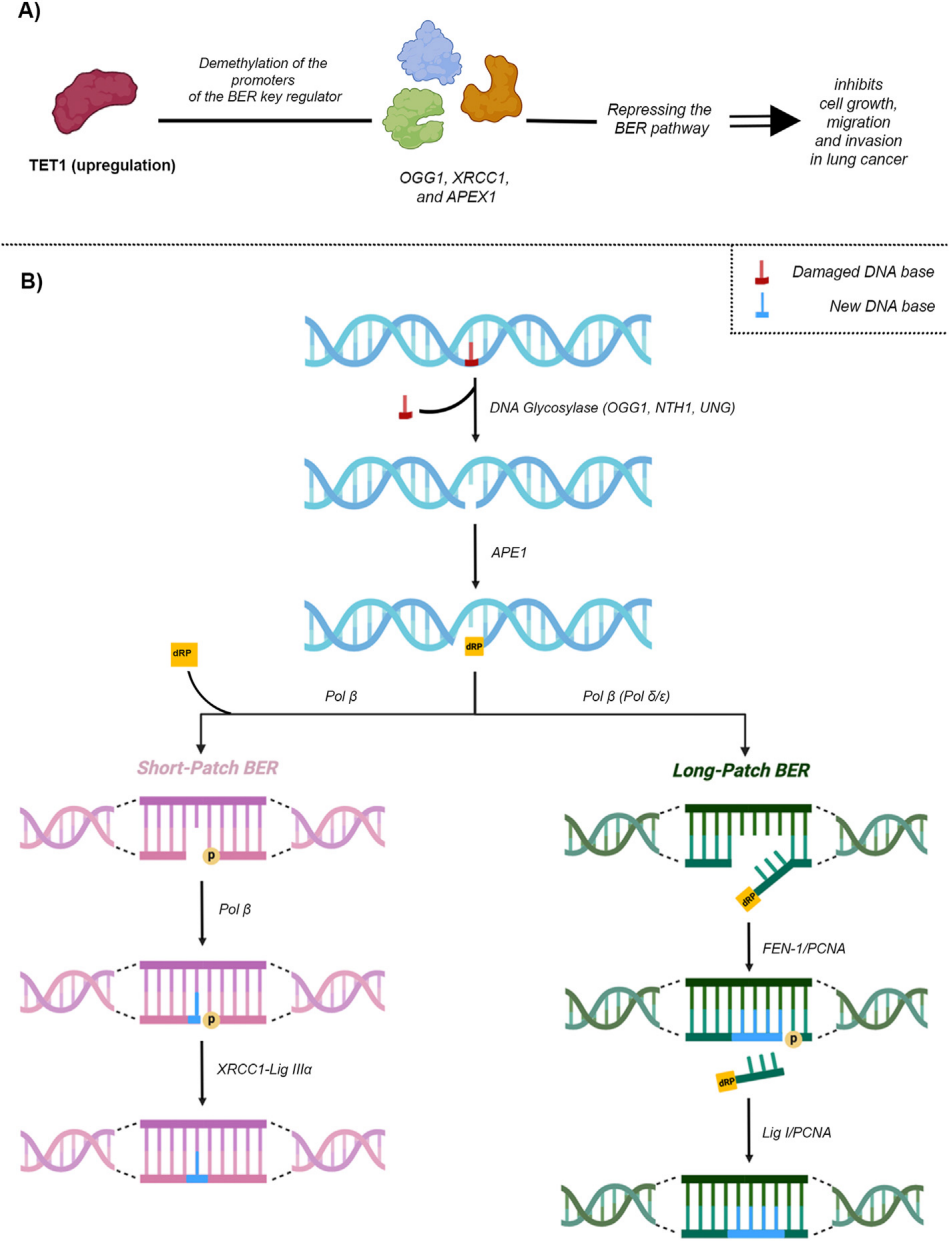
TET1-mediated BER pathway repression in lung cancer progression. This figure illustrates TET1's role in modulating lung cancer phenotype through regulation of the base excision repair (BER) pathway. **(A)** TET1 up-regulation induces demethylation of key BER regulator promoters, including OGG1, XRCC1, and APEX1, leading to repression of the BER pathway and inhibition of metastasis in lung cancer. **(B)** The diagram provides an overview of the BER excision repair mechanism, highlighting the roles of XRCC1 and OGG1, and their connection to TET1.

Recent studies have also linked TET1 to broader epigenetic mechanisms and highlighted potential therapeutic targets. Chen et al^60^ demonstrated that TET1 regulates DNA methylation and hydroxymethylation within the base excision repair pathway, particularly impacting genes like X-ray repair cross complementing 1 (XRCC1), 8-oxoguanine DNA glycosylase 1 (OGG1), and apurinic/apyrimidinic endonuclease 1 (APEX1). TET1 maintains hydroxymethylation at the promoters of these genes, and its loss has been associated with increased tumor growth, migration, and invasion (Fig. 3). In 2022, Xu et al^61^ identified loss-of-function mutations in TET genes in 7.4% of lung adenocarcinoma cases. These mutations frequently co-occurred with Kristen rat sarcoma (KRAS) mutations, resulting in poorer patient survival and significant epigenetic dysregulation of the Wnt signaling pathway. Notably, restoring TET activity or targeting β-catenin effectively inhibited tumor progression. Similarly, Alrehaili et al^62^ reported that decreased 5hmC levels and reduced TET expression in non-small cell lung cancer tissues were associated with adverse clinical features, suggesting that 5hmC levels could serve as potential prognostic biomarkers. Collectively, these findings highlight TET1's multifaceted role in lung cancer biology and its potential as a therapeutic and diagnostic target.

### Pancreatic cancer

As Wu et al^63^ outlined in 2019, TET1 acts as an inhibitor of pancreatic tumor progression and metastasis both *in vivo* and *in vitro* by suppressing Wnt signaling pathways. TET1 directly interacts with the promoter of secreted frizzled-related protein 2 (SFRP2), facilitating demethylation to activate SFRP2 transcription. This activation inhibited both canonical and noncanonical Wnt signaling pathways, ultimately impeding the epithelial–mesenchymal transition in pancreatic tumors (Fig. 2). Pancreatic cancer patients with low TET1 expression levels have poorer overall survival compared with those with higher TET1 levels. TET1 has been shown to overcome chemotherapy resistance in pancreatic ductal adenocarcinoma by reducing the activity of the cell adhesion molecule L1 like (CHL1)-associated Hedgehog signaling pathway. Consequently, patients with elevated TET1 levels tend to have a more favorable prognosis in pancreatic ductal adenocarcinoma cases.^64^ A research project by Li et al^65^ in 2022 underscored the crucial role of TET1-mediated demethylation in establishing β-cell identity. The study revealed that TET1 physically interacted with the transcription factor forkhead box A2 (FOXA2) to regulate pancreas development. Disrupting TET1, TET2, and TET3 genes led to significant defects in β-cell formation. Detailed analysis identified hypermethylated DNA regions enriched with FOXA2, emphasizing its importance in pancreatic development. TET gene depletion disrupted FOXA2 binding during the formation of pancreatic progenitors. Notably, introducing full-length TET1 restored β-cell differentiation and corrected paired box 4 (PAX4) methylation, highlighting a potential therapeutic avenue for pancreatic dysfunction.

### Prostate cancer

TET1 suppresses prostate cancer invasion by activating TIMPs.^30^ In a study focusing on high-risk prostate cancer, researchers investigated the genomic alterations contributing to the disease's progression. They found that TET1 protein levels were consistently lower in tumor tissues compared with non-tumor tissues, indicating TET1's significant role in preventing prostate cancer invasion. The clinical significance of these findings was validated in a separate prostate cancer cohort, where reduced TET1 mRNA levels were associated with poorer metastasis-free survival. The study also identified a substantial decrease in hydroxymethylated DNA within tumor tissues, highlighting TET1's crucial role in DNA modification and its potential impact on prostate cancer progression.^66^ Another study explored cell progression following the development of resistance to the prostate cancer drug enzalutamide. Researchers observed that low levels of a circular RNA, circUCK2, were linked to enzalutamide resistance. Increasing circUCK2 levels made miR-767-5p resistant to enzalutamide, which led to higher TET1 protein expression and subsequently inhibited cell invasion and proliferation. In contrast, reducing circUCK2 in sensitive cells lowered TET1 levels, thereby promoting cell invasion and proliferation. In mouse models, high circUCK2 expression inhibited the growth of enzalutamide-resistant prostate cancer cells. This research provides valuable insights for potential therapies aimed at better controlling the progression of castration-resistant prostate cancer.^67^ TET1 activation in prostate cancer also triggers the expression of genes involved in chromatin remodeling, mitosis, anti-viral processes, and stem cell pluripotency, with many of these genes showing associated promoter hypomethylation. Notably, a significant portion of these genes encode zinc finger transcription factors, which contain binding sites for TET1 and are coactivated by it.^68^

### Colorectal cancer

Frequent DNA methylation abnormalities are observed in colorectal cancer. Studies have shown that the TET1 gene is down-regulated during the early stages of colon tumorigenesis. Silencing TET1 in normal colon cells induces cell proliferation, while reintroducing it into colon cancer cells inhibits their growth, even at advanced stages. TET1 was found to interact with the promoters of DKK genes, which inhibit the Wnt pathway, maintaining them in a low-methylation state that enables their activation. In colon cancer, reduced TET1 levels lead to the repression of DKK genes through DNA methylation, resulting in the persistent activation of the Wnt–β-catenin signaling pathway. Reintroducing TET1 into colon cancer cells increases the expression of DKK genes, suppresses the Wnt–β-catenin pathway, and inhibits cell proliferation and tumor growth (Fig. 2).^69^ Findings suggest that TET1 plays a complex role in the development of colorectal cancer, influencing various aspects of tumor progression. In one study, researchers observed a significant reduction in TET1 expression in colorectal cancer tissues compared with normal tissues. Additionally, *in vitro* experiments demonstrated that TET1 could inhibit cell proliferation while promoting cell metastasis and invasion.^70^ In 2017, researchers emphasized the crucial role of TET1 in regulating E-cadherin and its impact on colon cancer progression. When TET1 is reduced or knocked down, it triggers epithelial–mesenchymal transition, leading to increased cancer cell migration, invasion, and growth. This reduction in TET1 also results in elevated EZH2 expression and decreased UTX-1 expression, which in turn represses the E-cadherin gene. However, restoring UTX-1 expression or depleting EZH2 counteracts these effects by suppressing epithelial–mesenchymal transition and inhibiting tumor invasion in colon cancer cells.^71^ In 2018, researchers highlighted the intricate interplay between epigenetic mechanisms and the tumor microenvironment, particularly in response to hypoxia, and its impact on colorectal cancer progression. Their findings reveal that hypoxia increases TET1 expression through a mechanism involving hypoxia-inducible factor 1-alpha (HIF-1α). TET1, in turn, influences gene expression by binding to hypoxia-responsive elements (HREs), with this interaction being modulated by CpG methylation levels. Notably, while the loss of TET1 does not affect cell proliferation, it significantly inhibits cell migration, which is a crucial aspect of cancer progression. Additionally, the study identified specific TET1 gene mutations, such as E2082K, that counteracted its pro-migratory effects.^72^ In a research effort by Ma et al,^73^ researchers emphasized TET1's role as a tumor suppressor in colorectal cancer and proposed that miR-21 may contribute to colorectal cancer development by down-regulating TET1. They discovered that miR-21, a gene regulator, was up-regulated in colorectal cancer tissues, while TET1, known to inhibit cell growth, was down-regulated. Notably, miR-21 was found to negatively correlate with TET1 expression. When miR-21 levels were reduced using inhibitors, TET1 expression increased. Further analysis revealed that miR-21 promoted colorectal cancer cell proliferation by targeting and down-regulating TET1. A similar study by Cheng et al^74^ examined tumor samples from colorectal cancer patients and found an association between reduced TET1 levels, down-regulated by miR-21-5p, and the progression of colorectal cancer to more advanced stages. Additionally, dietary compounds have been shown to play an anti-cancer role by influencing DNA demethylation mechanisms. In a study by Kang et al,^75^ the researcher focused on luteolin, a dietary flavone, and its ability to promote apoptosis in human colon cancer cells. They have discovered that luteolin promotes DNA demethylation of the Nrf2 promoter, which increases Nrf2 expression and enhances its interaction with the tumor suppressor p53. Additionally, luteolin inhibits DNMTs, thereby boosting the activity of TET DNA demethylases such as TET1. This results in reduced methylation of the Nrf2 promoter, which correlates with an increase in Nrf2 expression. Additionally, luteolin enhances TET1 binding to the Nrf2 promoter, while knockdown of TET1 reduces the apoptosis induced by luteolin. These findings highlight luteolin's anti-cancer effects in colon cancer, suggesting a link between Nrf2 up-regulation, p53 interaction, and TET1-mediated DNA demethylation. In a study by Yang et al,^76^ the critical role of TET1 in regulating the methylation level of the zinc-finger protein ZNF334 was highlighted. Aberrant DNA methylation, a hallmark of colorectal cancer development, was found to affect ZNF334, with TET1 identified as a key regulator of this process. Researchers developed a targeted demethylation system utilizing TET1 and sgRNA to reverse methylation in the ZNF334 promoter. Their experiments, conducted both *in vitro* and *in vivo*, demonstrated that this approach successfully restored ZNF334 expression, leading to the suppression of colorectal cancer growth.

### Glioma and glioblastoma

In gliomas, alterations in the Isocitrate dehydrogenase 1 (IDH1) gene play a pivotal role in tumor formation. These mutations result in the production of 2-hydroxyglutarate, an oncometabolite that inhibits the activity of oxygenases, including TET enzymes, which are essential for converting 5mC to 5hmC. A previous study revealed a strong correlation between IDH1 mutations and the nuclear localization of TET1 in glioma cells. In cases where 5hmC was absent, 70 % of gliomas showed either exclusive or predominant cytoplasmic expression of TET1 or lacked detectable TET1 protein altogether.^77^ In a research project led by Kuhns et al,^78^ the role of TET1 in DNA damage response and repair in glioblastoma was investigated. Suppressing TET1 in glial cells resulted in reduced sensitivity to ionizing radiation, although no change in sensitivity was observed with inhibitors of Na^+^/K^+^-ATPase. TET1-deficient glial cells exhibited decreased markers of DNA damage response, such as γH2A.x and 53BP1 foci, in response to ionizing radiation and topoisomerase inhibitors. Notably, TET1 deficiency resulted in increased DNA strand breaks and reduced DNA repair efficiency, suggesting a link between TET1 and genomic instability. This impaired DNA repair may contribute to the poor prognosis associated with glioblastomas. Another study confirmed TET1's role as a tumor suppressor and further elucidated the molecular mechanisms regulated by TET1 in gliomas. The study showed that reduced TET1 levels stimulated glioma cell proliferation, migration, and invasion. This was accompanied by an increase in the expression of β-catenin, as well as downstream molecules such as cyclin D1 and c-Myc. Rescue experiments revealed that down-regulating β-catenin significantly reduced glioma cell proliferation both *in vivo* and *in vitro*.^79^

### Cervical cancer

Studies suggest that decreased TET1 expression may play a crucial role in the development and progression of cervical cancer. In a study, researchers examined the expression levels of TET1 proteins in cancerous and non-cancerous cervical tissues. The study found a significant reduction in TET1 transcript levels in cervical tissue samples from patients with primary cervical cancer compared with those from control patients. Low TET1 expression was associated with various clinicopathological variables, including cancer stage, grade of differentiation, patient age, and histological type.^80^ TET1 is emerging as a key player in the pathogenesis of cervical cancer. Its expression levels change significantly as the disease progresses, peaking in high-grade lesions and decreasing in invasive cancer. TET1 is associated with stemness properties in high-grade lesion cells, potentially influencing cancer development. Importantly, TET1 inhibits epithelial–mesenchymal transition, a process linked to cancer progression and metastasis. It also directly regulates gene expression through epigenetic modifications, impacting critical genes such as zinc finger E-box binding homeobox 1 (ZEB1) and vimentin (VIM).^81^ In a recent study by Ren et al,^82^ researchers observed that TET1 expression was significantly reduced in cervical cancer tissues. Suppressing TET1 in cervical cancer cells led to increased invasion, cell proliferation, migration, self-renewal, and tumor formation. Conversely, overexpressing TET1 reversed these processes. Additionally, TET1 influenced autophagy levels in cervical cancer cells by mediating the methylation of autophagy promoter regions, particularly in genes like nuclear factor-κB repressing factor (NKRF) and histone H2A type 1 (HIST1H2AK). Moreover, TET1 plays a complex role in the interplay between human papillomavirus infection and cervical cancer progression. As highlighted in a recent study by Dutta et al,^83^ researchers examined the role of epigenetic modifier genes DNMT1 and TET1 in cervical cancer in the context of human papillomavirus infection. Using bioinformatics, genetic profiling, and epigenetic analysis, they correlated their findings with clinical parameters. The study revealed that human papillomavirus infection disrupts the epigenetic balance of DNMT1 and TET1, leading to increased DNMT1 expression and decreased TET1 expression during cervical cancer development. Low DNMT1 methylation, along with high TET1 promoter methylation and deletion frequencies, contributes to their antagonistic expression. The promoter methylation of these genes in plasma DNA may have diagnostic value, and molecular alterations in TET1, either alone or in combination with DNMT1, are associated with poor patient survival.

### Hepatocellular carcinoma and cholangiocarcinoma

Researchers have discovered that TET1 expression is reduced in hepatocellular carcinoma (HCC) tissues, indicating its potential role as a tumor suppressor. It was demonstrated that miR-29b inhibited metastasis by targeting TET1, suggesting that decreased miR-29 expression may contribute to HCC development and progression through the up-regulation of TET1.^84^ Additionally, miR-494^85^ and miR-520b^86^ were found to promote tumor vascular invasion and suppress proliferation in liver cancer cells, respectively, by targeting TET1. In a study by Chen et al,^87^ they investigated the influence of cyclooxygenase-2 (COX-2) on HCC using transgenic mice with liver-specific COX-2 overexpression. A significant finding was the central role of the DNA demethylase enzyme TET1 in HCC development. Elevated COX-2 expression in hepatocytes resulted in spontaneous HCC formation in the transgenic mice, along with increased inflammation and blood vessel formation within the tumors. Crucially, the study revealed that COX-2-induced HCC was associated with DNA promoter hypermethylation, driven by reduced TET1 expression. This led to the loss of an essential DNA modification, 5hmC, and the down-regulation of tumor-suppressive genes, including latent transforming growth factor beta-binding protein 1 (LTBP1). Further research by Liu et al^88^ demonstrated the effectiveness of transiently transfecting HCC cells with the TET1 catalytic domain. This transfection significantly reduced the methylation levels of tumor suppressor genes such as Ras association domain family member 1A (RASSF1A), adenomatous polyposis coli (APC), p16, TET1, and suppressor of cytokine signaling 1 (SOCS1), while increasing the expression levels of these tumor suppressor genes. Consequently, HCC cell proliferation, migration, and invasion were suppressed. Importantly, this effect was specific to tumor suppressor genes, as the methylation and transcript levels of oncogenes remained unchanged. TET1 can also contribute to promoting cell growth in HCC. In a 2021 study, researchers analyzed clinical liver samples and identified a subgroup of HCC cases with elevated TET1 expression, which exhibited gene expression patterns similar to those of liver progenitor cells. They also observed abnormal DNA modifications (5hmC) primarily in active enhancer regions of the genome. When TET1 expression was reduced in hepatoma cell lines, cell growth was suppressed. Furthermore, the study highlighted the oncogene HMGA2, which was highly expressed in a subgroup of HCC cases with elevated TET1 levels. This expression was linked to specific epigenetic changes that promoted cell proliferation.^89^ In addition, TET1 is also involved in the autophagy process of HCC. According to a study by Sun et al,^90^ increasing TET1 levels in HCC cells inhibits cancer cell growth, migration, invasion, and inflammation while promoting autophagy and apoptosis. TET1 activates miR-34a through demethylation, which then targets the BTB domain and CNC homolog 1 (BACH1). BACH1 negatively regulates the p53 pathway. This interconnected network involving TET1, miR-34a, BACH1, and p53 plays a crucial role in HCC progression. The role of TET1 in cholangiocarcinoma was investigated in 2021 by Bai et al.^91^ The study demonstrated that TET1, which depends on 2-oxoglutarate (OG), is significantly up-regulated in cholangiocarcinoma patients. Experiments using OG-related compounds revealed that TET1 promoted cholangiocarcinoma cell malignancy by increasing 5hmC levels. Inhibiting TET1 activity through various methods suppressed cholangiocarcinoma development by affecting cell growth and apoptosis. Moreover, TET1 appears to be a marker of HCC progression, influencing immune responses and oncogenic pathways. In a recent study by Wen et al,^92^ researchers found that TET1 expression is significantly higher in tumor samples of HCC compared with normal samples. Patients with advanced stages and higher grades of HCC exhibited elevated TET1 expression, which correlated with a worse prognosis. Notable differences were observed in immune cell infiltration and responses to immunotherapy and chemotherapy between groups with high and low TET1 expression. The study also identified 90 genes associated with DNA demethylation that showed differential expression based on TET1 levels. A predictive model was developed using these genes, incorporating seven key prognostic markers (UGT2B15, SERPINH1, CYP2C9, SPHK1, CDC20, SLC1A5, and HACD2), which effectively predicts HCC outcomes.

### Inflammatory disorders

The role of TET1 in childhood asthma and allergic airway inflammation was investigated in 2019. Researchers found that TET1 deficiency exacerbated the severity of the disease, leading to increased airway hyperresponsiveness and lung eosinophilia. TET1-deficient mice exhibited elevated expression of inflammation-related genes. Transcriptomic analysis revealed up-regulation of the interferon (IFN) signaling pathway and down-regulation of the aryl hydrocarbon receptor pathway in TET1-deficient mice, with similar effects observed in human bronchial epithelial cells. These changes were associated with alterations in DNA methylation, transcription factor binding, and histone modifications linked to TET1.^93^ Moreover, TET1 can play a role in promoting inflammation by modifying DNA methylation patterns in response to hypoxia in adipocytes. In a research project led by Ali et al,^94^ they hypothesized that hypoxia-induced up-regulation of hypoxia-inducible factor HIF1α could influence DNA methylation patterns and, in turn, affect the expression of inflammatory adipocytokines. They investigated the potential involvement of the DNA demethylase TET1, which is known to be induced by HIF1α. Their experiments in human adipocytes showed that hypoxia increased both HIF1α and TET1 levels, resulting in global hydroxymethylation, reduced DNA promoter methylation of adipocytokines, and enhanced adipocytokine expression. Importantly, these effects were alleviated by HIF1α inhibition or TET1 gene silencing. The study identified key hypoxia-responsive adipocytokines, including leptin, interleukin 6 (IL6), interleukin 1 beta (IL1β), tumor necrosis factor-alpha (TNFα), and interferon-gamma (IFNγ). In a recent study by Tricarico et al,^95^ researchers investigated the role of TET1 in inflammation using ApcMin mice as a model. They observed that mice expressing the TdgN151A allele had an increased number of small intestinal adenomas. In contrast, Tet1-deficient and Tet1/TdgN151A double heterozygous ApcMin mice developed larger colonic adenomas with invasive characteristics. Further analysis revealed that colonic adenomas in Tet1 and Tdg mutant ApcMin mice exhibited reduced global DNA hypomethylation. Notably, Tet1-mutant adenomas displayed hypermethylation of CpG islands. These genetic variations were linked to the up-regulation of genes associated with inflammation, immune responses, and interferon pathways in Tet1 and Tdg-mutant colonic adenomas, compared with control adenomas. The study also identified a 127-gene inflammatory signature that categorized colonic adenocarcinomas into distinct groups based on genomic instability, DNA methylation levels, and DNMT1 expression. Interestingly, tumors with the CpG island methylator phenotype exhibited high DNMT1 and low TET1 expression.

### Autoimmune diseases

TET1 plays a critical role in autoimmune diseases by regulating the function and differentiation of regulatory T cells, which are essential for immune homeostasis. Along with TET2, TET1 is maintained by hydrogen sulfide and participates in converting 5mC to 5hmC in the forkhead box P3 (Foxp3) gene. This conversion ensures a specific DNA methylation pattern that stabilizes Foxp3 expression in regulatory T cells. Loss of TET1 and TET2 leads to hypermethylation of Foxp3, impairing regulatory T cell differentiation and function, ultimately contributing to the development of autoimmune diseases.^96^ In another study conducted in 2022, TET1's role in autoimmune disease was investigated in the context of systemic sclerosis. When TET1 is overexpressed due to the activation of 2′-5′-oligoadenylate synthetase-like (OASL) and interferon regulatory factor 1 (IRF1) signaling, TET1 increases the hydroxymethylation levels of CD4^+^ T cells. This epigenetic modification may contribute to the aberrant activation of CD4^+^ T cells and the overexpression of functional proteins, thereby promoting immune dysfunction in autoimmune diseases such as systemic sclerosis.^97^

### Cardiovascular diseases

TET1 appears to play a protective role in cardiovascular disease, particularly in atherosclerosis. According to research by Cao et al,^98^ aberrant expression of TET1 and H19 lncRNA is observed in endothelial cells of atherosclerotic coronary arteries. When exposed to TNFα, a known risk factor for cardiovascular disease, H19 expression is increased, subsequently activating transforming growth factor-beta (TGF-β) signaling and promoting endothelial-to-mesenchymal transition. In addition, TET1 is essential in this epigenetic process. H19 also regulates TET1 expression post-transcriptionally. The H19/TET1 axis likely plays a crucial role in endothelial-to-mesenchymal transition and may contribute to the development of cardiovascular disease, presenting a potential therapeutic avenue for further investigation. In another study by Qu et al^99^ in 2022, the deletion of TET1 in mice led to more severe atherosclerotic lesions throughout the aorta compared with mice with a different TET1 mutation. These lesions were particularly concentrated in areas exposed to oscillatory shear stress, such as the aortic arch and abdominal aorta. Additionally, TET1 was found to play a role in maintaining the vascular intimal barrier and enhancing vascular endothelial barrier function by regulating gene expression, particularly the connexin-40 (CX40) gene, through histone acetylation.

### Reproductive and developmental disorders

The regulation of reproduction by TET1 may involve its influence on the expression of gonadotropin hormones essential for follicle development, maturation, and ovarian function. Researchers have found that TET1 expression decreases as cell differentiation progresses, with poorly differentiated gonadotropes expressing a TET1 isoform lacking a specific domain. This isoform directly represses luteinizing hormone gene expression, independent of 5hmC catalysis at its promoter.^100^ TET1 also plays a crucial role in regulating aging in spermatogonia stem cells and reproductive health. In 2020, Huang et al^101^ demonstrated that Tet1 deficiency in mice led to a progressive reduction in spermatogonia stem cells, impaired spermatogenesis, and accelerated infertility with age. This deficiency results in decreased levels of 5hmC and down-regulation of genes critical for cell cycle regulation, germ cell differentiation, meiosis, and reproduction. TET1 also regulates signaling pathways related to stem cell development, including the Wnt and PI3K-Akt pathways, as well as stress response genes and autophagy. In contrast, the impact of TET2 deficiency on male reproductive aging is comparatively limited. Similarly, Liu et al^102^ found that TET1-deficient mice experienced a dramatic reduction in their follicle reserve, resembling premature ovarian failure syndrome. These mice became infertile in middle age, while wild-type mice remained reproductively viable. TET1 deficiency results in molecular changes in oocytes, including altered organelle function, disrupted ubiquitination and autophagy, and modifications in signaling pathways associated with Alzheimer's disease. It also affects the expression of X-chromosome linked genes, such as fragile X messenger ribonucleoprotein 1 (Fmr1), which are associated with premature ovarian failure. Additionally, TET1 deficiency leads to abnormal expression of long interspersed nuclear element-1 (LINE-1) and endogenous retroviruses in oocytes, consistent with oocyte senescence and follicle atresia observed in premature ovarian failure.

### Neurological disorders

In 2013, scientists proposed that TET1 may have a crucial role in cognitive function. Their findings indicate that TET1 is significant in regulating neural progenitor cell proliferation in the adult mouse brain. The absence of TET1 in mice results in impaired hippocampal neurogenesis and poor learning and memory.^103^ TET1's role in the context of schizophrenia and bipolar disorder is complex and may involve mechanisms that are independent of its DNA hydroxymethylation activity. It has been discovered that specific genes associated with these disorders are down-regulated, with overexpression of DNMT1 and TET1 observed. However, increased DNMT1 binding to gene promoters in the cortex is detected, which does not always correlate with increased DNA methylation. This suggests a region-specific and potentially neuron-activity-dependent mechanism that influences gene expression in these neurological disorders.^104^ Despite ongoing uncertainty regarding the reliability of peripheral tissues, such as blood and saliva, as biomarkers for neuropsychiatric disorders, a study using blood samples from individuals experiencing their first episode of psychosis identified a connection between elevated levels of TET1, neuregulin 1 (NRG1), brain-derived neurotrophic factor (BDNF), and DNMT1, and the onset and progression of schizophrenia.^105^ TET1 is also implicated in behavioral deficits and has been shown to regulate an epigenetic program associated with myelination, calcium transport, and cell division. The study identifies a shift in DNA hydroxymethylation patterns during the differentiation of oligodendrocyte progenitor cells and highlights the importance of TET1 in proper oligodendrocyte development, myelination, and remyelination in the mouse brain.^106^ Recently, scientists investigated the role of TET1 in Alzheimer's disease by examining TET1 mutations in individuals with early-onset Alzheimer's disease and identified significant associations. Additionally, a mouse model with TET1 knockout was used, revealing increased Alzheimer's disease-related pathology, including amyloid plaque burden and changes in gene expression. These findings suggest that TET1 may contribute to the pathogenesis of neurological disorders, including Alzheimer's disease, through its influence on epigenetic modifications and gene expression.^107^

### Metabolic disorders

Wang et al^108^ found that TET1 played a protective role against non-alcoholic fatty liver disease by promoting fatty acid oxidation through the activation of the peroxisome proliferator-activated receptor alpha (PPARα) pathway. TET1 was found to bind directly to the promoter of PPARα and increase its hydroxymethylation level, ultimately inhibiting the progression of non-alcoholic fatty liver disease.^108^ Further studies highlight TET1's critical role in adipocyte differentiation from mesenchymal stem cells. TET1 was found to be up-regulated during differentiation, and its inhibition led to a decrease in adipocyte differentiation. Additionally, depletion of TET1 hindered the gain of 5hmC and impaired adipocyte differentiation. Analysis of gene expression and epigenetic modifications revealed that TET1 knockout resulted in the down-regulation of genes associated with lipid metabolism and fat cell differentiation. One key gene affected by TET1 modulation was retinoid X receptor alpha (Rxra), with studies suggesting that Rxra is essential for adipogenesis.^109^ TET1 also plays a fundamental role in regulating glucose metabolism, with implications for the treatment of metabolic disorders. In 2021, Zhang et al^110^ have revealed that TET1 interacts with silent information regulator T1 (SIRT1) in the liver and activates its deacetylase activity. This activation, in turn, regulates the acetylation-dependent translocation of transcription factors such as peroxisome proliferator-activated receptor-γ coactivator-1α (PGC-1α) and forkhead box O1 (FOXO1). As a result, hepatic gluconeogenic gene expression, which is critical for glucose metabolism, is activated. Importantly, fasting-induced gene activation is inhibited in mice with reduced TET1 levels. Additionally, another study demonstrated that AMP-activated protein kinase (AMPK) activators, which mimic fasting, enhanced hepatic gluconeogenic gene expression through the AMPK–TET1–SIRT1 axis. The role of TET1 in obesity was further illustrated in 2022. The scientists have revealed that TET proteins act as epigenetic suppressors of the β3-adrenergic receptor (β3-AR) in adipocytes. When all three TET genes are deleted in adipocytes, β3-AR expression increases, leading to enhanced responses to β-adrenergic stimulation. This results in increased lipolysis, activation of thermogenic genes, fat browning, and enhanced oxidative metabolism. Notably, mice with adipose-specific TET gene ablation maintain higher β3-AR levels, remain responsive to β-adrenergic stimuli even on a high-fat diet, and exhibit improved energy expenditure, reduced fat accumulation, and protection from diet-induced obesity and related metabolic complications. Mechanistically, TET proteins repress β3-AR transcription, primarily through an enzymatic activity-independent mechanism involving histone deacetylases.^111^ TET1 has also been identified as a significant epigenetic regulator specifically targeting beige adipocytes to suppress the thermogenic gene program, offering potential therapeutic avenues for enhancing energy expenditure in metabolic disorders like obesity. In 2020, researchers have shown that TET1 expression in subcutaneous adipose tissue is suppressed by cold and other stimuli that activate beige adipocyte thermogenesis. TET1 acts as a powerful repressor of key thermogenic genes in beige adipocytes. To explore its effects further, adipose-specific TET1 knockout mice are generated, resulting in improved energy expenditure, increased cold tolerance, and protection against diet-induced obesity and insulin resistance. Crucially, TET1's role in inhibiting thermogenic genes is largely independent of its DNA demethylase activity. Instead, TET1 cooperates with histone deacetylase 1 (HDAC1) to induce epigenetic changes that repress thermogenic gene transcription.^112^

## Application of TET inhibitors for treating the TET1-altered diseases

The development of TET inhibitors has garnered significant attention due to the pivotal role of TET enzymes, particularly TET1, in various diseases. Researchers have explored various approaches, including small molecule inhibitors, peptide-based strategies, and combination therapies, to inhibit TET1 activity for therapeutic purposes. Promising small molecule inhibitors include 2-hydroxyglutarate analogues, divalent metal chelators, and nucleoside analogues. Additionally, peptide-based inhibitors targeting the CXXC domain of TET1 have shown potential in disrupting its DNA binding.^113^^,^^114^ These inhibitors are being investigated for use in TET1-altered diseases, including breast cancer,^115^ acute myeloid leukemia,^44^ and T-cell acute lymphoblastic leukemia.^116^ They have shown promising results in reducing cell viability, affecting tumor growth pathways, and reversing disease progression. Despite these advances, challenges remain, such as achieving specificity, effective delivery, and identifying reliable biomarkers. Future research aims to address these hurdles and refine TET inhibition strategies, paving the way for more targeted and personalized treatments for TET1-related conditions.

## Conclusions

In the realm of epigenetic modification, DNA demethylation is crucial, and TET1 plays a central role in this intricate process. While advanced studies have produced promising results, certain critical aspects remain shrouded in ambiguity, necessitating further investigation. Numerous studies have identified various factors involved in regulating TET enzyme functionality, including transcription factors, microRNAs (as summarized in Table 1), post-translational modifications, and small molecules. The complex network surrounding TET1 requires deeper exploration to fully understand the underlying mechanisms. The enigmatic role of TET1 in tumorigenesis remains a subject of intense debate, as its impact exhibits a dual nature. The inactivation of tumor suppressor genes via CpG island promoter hypermethylation is a well-established oncogenic mechanism. In many tumors, TET1 acts as an “activator” mitigating hypermethylation-induced gene silencing. Yet, emerging research presents a more complex narrative, revealing that TET1 displays variable expression across different tumor types and subtypes, triggering distinct biological effects by regulating diverse genes in various signaling pathways. This duality, encompassing both oncogenic and tumor-suppressive properties, underscores the need for a deeper understanding of the underlying mechanisms (as detailed in Table 2).

**Table 1:** MicroRNAs interacting with TET1 and their association with various diseases.

MicroRNA	Expression	Role in regulating TET1	Disease association	Regulatory mechanism	Reference
miR-21	Upregulated	Downregulates TET1	Colorectal cancer	miR-21-5p negatively correlates with TET1 expression, targets TET1 to promote cell growth, promotes CRC cell proliferation, diagnostic and prognostic biomarker in CRC	73, 74
miR-21	Upregulated in HCC exosomes	Downregulates PTEN and PTENp1 expression	Hepatocellular carcinoma (HCC)	Positively correlated with miR-21 expression in cells; negatively correlated with PTEN, PTENp1, and TETs expression; promotes HCC cell proliferation, migration, and inhibits apoptosis; increases methylation level of PTENp1 promoter through regulating TETs expression.	^117^
miR-21	Highly expressed in BMSCs-derived EVs	Downregulates TET1 expression	Rheumatoid arthritis (RA)	Targets TET1, leading to alleviation of RA by promoting the TET1/KLF4 regulatory axis.	^118^
miR-27a-3p	Upregulated	Downregulates TET1	Breast cancer	• Direct binding to TET1 3′UTR • Regulates EMT • Promotes proliferation, migration, and invasion of breast cancer cells	^119^
miR-29	Upregulated	Downregulates TET1	Breast cancer	• miR-29a acts as a tumor activator by targeting TET1 in breast cancer. • miR-29b/TET1/ZEB2 signaling axis has been identified as a key regulator of metastatic properties and EMT	120, 121
miR-29	Upregulated	Downregulates TET1	Lung cancer	Regulated by MAPK-driven ETS1 transcription factor, leading to reduced 5hmc epigenetic modifications	^55^
miR-29	Downregulated	Inhibits metastasis by targeting TET1	Hepatocellular carcinoma (HCC)	Contributes to the malignant progression of the disease	^84^
miR-29	Downregulated	Downregulates TET1	Acute myeloid leukemia	Acts as a tumor suppressor in leukemogenesis by regulating TET1 expression.	^122^
miR-29	Upregulated	Downregulates TET1	Colorectal cancer	miR-29a-5p is the key player in the development of colitis-associated colorectal cancer (CAC), with its overexpression leading to decreased levels of TETs and 5hmC	^123^
miR-543	Upregulated in nonresponders to ruxolitinib therapy	Downregulates both TET1 and TET2	Myelofibrosis (MF)	Targets the dioxygenases TET1 and TET2, causing increased levels of global 5-methylcytosine; decreases the acetylation of histone 3, STAT3, and tumor protein p53; activated by STAT3 via epigenetic control; promotes the expression of genes related to drug metabolism, including CYP3A4, involved in ruxolitinib metabolism.	^124^
miR-26a	Downregulated	Downregulates TET1 expression	MLL-rearranged acute myeloid leukemia (AML)	Functions as a tumor-suppressor mediator; transcriptional repression by c-Myc; restoration of miR-26a expression/function holds therapeutic potential to treat MLL-rearranged AML.	^122^
miR-22	Repressed in AML	Upregulates TET2 and TET3 expression; downregulates TET1 expression	Acute myeloid leukemia (AML)	Epigenetic repression of miR-22 transcription mediated by TET1, SIN3A, and EZH2; miR-22 locus affected by DNA copy loss in 8–20 % of AML cases; negatively regulates critical oncogenes CRTC1, FLT3, and MYCBP	^125^
miR-200s	Downregulated	Correlated with TET1 expression	Lung adenocarcinoma (LUAD)	Interacts with MBD2-TET1 complex, critical regulators of tumor invasiveness; potential therapeutic intervention targets.	^58^
miR-494	Upregulated in HCC tumors with vascular invasion	Downregulates TET1 expression	Hepatocellular carcinoma (HCC)	Direct targeting of TET1 by miR-494 triggers gene silencing of multiple invasion-suppressor miRNAs; reduced 5′-hydroxymethylcytosine levels in CpG regions of invasion-suppressor miRNA genes associated with their transcriptional repression	^85^
miR-520b	Downregulated	Downregulates TET1 expression	Hepatocellular carcinoma (HCC)	Suppresses cell proliferation in HCC through targeting the 3′-untranslated region (3′UTR) of TET1 mRNA; reduces the expression of TET1 at the levels of mRNA and protein; inhibits proliferation of hepatoma cells, but TET1 overexpression rescues the inhibition of cell proliferation mediated by miR-520b; expression levels of miR-520b negatively related to those of TET1 mRNA in clinical HCC tissues.	^86^
miR-34a	Upregulated in response to TET1 activation	Upregulates TET1 expression	Hepatocellular carcinoma (HCC)	Activated by TET1 through demethylation; targets BACH1, a negative regulator of the p53 pathway; involved in inhibiting cancer cell growth, migration, invasion, and inflammation while promoting autophagy and apoptosis in HCC.	^90^
miR-30a	–	Downregulates TET1 expression	Idiopathic pulmonary fibrosis (IPF)	Inhibits TET1 expression through base pairing with complementary sites in the 3′ untranslated region (3′ UTR) to regulate Drp-1 promoter hydroxymethylation	^126^
miR-365a-3p	Highly expressed in Hep-2 cells, lowly expressed in BESB-2B cells	Downregulates TET1 expression	Laryngeal cancer	miR-365a-3p directly targets TET1, leading to decreased proliferation and invasion of Hep-2 cells *in vitro*.	^127^

**Table 2:** Dichotomous role of TET1 as oncogene or tumor suppressor in different cancer types.

Type of Cancer	Role	Mechanism/Effect	References
Breast cancer	Tumor suppressor	- Enhancing TET1 expression through EZH2 inhibition, leads to cell cycle arrest and senescence induction. (TNBC) - Increases LRIG1 expression through Tet1-dCas9 and VP64-dCas9, reducing cancer cell viability. (Basal/TNBC) - Inhibits Wnt/β-catenin pathway through MAGI2-AS3, reducing cell proliferation and migration. (BC)	36, 37, 38
Breast cancer	Oncogene	- Overexpression of TET1 in TNBC leads to hypomethylation of specific CpG sites, contributing to worse overall survival. (TNBC) - Associated with activation of oncogenic pathways such as PI3K, EGFR, and PDGF. - Correlates with sensitivity to drugs targeting the PI3K-mTOR pathway. (TNBC) - CRISPR-mediated deletion of TET1 in TNBC cell lines resulted in reduced expression of PI3K pathway genes, upregulation of immune response genes, and decreased cellular proliferation, suggesting a dependence of oncogenic pathways on TET1 overexpression. (TNBC)	^34^
Lymphoma	Tumor suppressor	- Prevents B cell lymphoma development in hematopoietic malignancies like diffuse large B-cell lymphoma (DLBCL) and follicular lymphoma (FL)[34]. - Loss of Tet1 has been associated with the development of B cell malignancies. - TET1 is hypermethylated and silenced in human B-NHL (B-cell non-Hodgkin lymphoma). - Mutations in genes related to histone modification and DNA methylation have been identified in Tet1-deficient tumors, further supporting its role as a tumor suppressor in lymphoma.	42, 46
Leukemia	Tumor suppressor/Dual role	- Represses miR-22 expression, affecting oncogenic pathways in acute myeloid leukemia (AML). - Exhibits dual roles in T-cell acute lymphoblastic leukemia (T-ALL): Promotes tumor growth when overexpressed, suppresses it when MYC is deactivated, and affects DNA methylation patterns and cell proliferation.	43, 49
Leukemia	Oncogene	- Targeted for inhibition in AML cases with elevated TET1 levels.	^44^
Lung adenocarcinoma	Tumor suppressor	- Acts as a tumor suppressor, with loss-of-function mutations associated with poor survival.	^61^
Non-small cell lung cancer	Tumor suppressor	- Reduced TET1 expression linked to unfavorable clinical features, suggesting potential prognostic value in NSCLC.	^62^
Pancreatic cancer	Tumor suppressor	- Functions as an inhibitor of Wnt signaling pathways in pancreatic cancer, contributing to tumor suppression. - Overcomes chemotherapy resistance, enhancing the efficacy of treatment. - Influences β-cell identity in pancreatic tumors, potentially impeding cancer progression.	^63^
Glioma	Tumor suppressor	- TET1 acts as a tumor suppressor in glioma by inhibiting cell proliferation, migration, and invasion, likely through the regulation of β-catenin and downstream molecules such as cyclinD1 and c-Myc.	^79^
Bladder cancer	Tumor suppressor	- TET1 downregulation increases cell proliferation and invasiveness in UBC cells, while restoring normal TET1 expression suppresses tumor growth in animal models.	^28^
Bladder cancer	Oncogene	- Decreased TET1 levels hinder hydroxymethylation of the AJAP1 promoter, activating β-catenin signaling and promoting UBC development. - OCT3/4 enhances the TET1-NRF2-MDM2 pathway, potentially contributing to tumor immune evasion in bladder cancer.	28, 29
Mixed-lineage leukemia-Rearranged leukemia (MLL)	Oncogene	- TET1 implicated as an oncogene in MLL-rearranged leukemia, promoting leukemogenesis and disease progression.	^122^
Prostate cancer	Tumor suppressor	- Suppresses invasion by activating TIMPs (Tissue inhibitors of metalloproteinases), which are involved in inhibiting the breakdown of the extracellular matrix, thus impeding cancer cell invasion. - In cases of high-risk prostate cancer (PCa), both TET1 mutations and decreased mRNA levels are linked to poorer outcomes in terms of metastasis-free survival.	30, 66
Cervical cancer	Tumor suppressor	- Affects autophagy levels in cervical cancer cells and mediates the methylation of autophagy promoter regions, particularly in genes NKRF and HIST1H2AK. - TET1 knockdown in cervical cancer cells promotes cell proliferation, self-renewal, migration, invasion, and xenograft growth, indicating its crucial role in inhibiting the migration and invasion of cervical cancer cells. - Directly regulates gene expression through epigenetic modifications, impacting key genes like ZEB1 and VIM. - TET1 expression peaks in high-grade lesions (HSIL) and decreases in invasive cancer.	81, 82
Colorectal cancer	Tumor suppressor	- Maintains hypomethylation of the DKK gene, inhibiting the WNT pathway, a critical pathway often dysregulated in colorectal cancer. - Re-expression of TET1 in colon cancer cells inhibits their proliferation and the growth of tumor xenografts, even at later stages. - TET1 is downregulated in colon tumors from the initial stage, and its silencing in primary epithelial colon cells increases cellular proliferation. - Regulates E-cadherin expression, suppressing epithelial–mesenchymal transition (EMT), tumor invasion, migration, and growth. - Downregulated by miR-21, promoting CRC cell proliferation. - Controls the methylation level of the zinc-finger protein ZNF334, impacting CRC growth.	69, 71, 73, 76
Thyroid cancer	Dual role	- TET1 serves as a suppressor of tumor growth in normal oxygen conditions (normoxia). Surprisingly, under conditions of low oxygen (hypoxia), TET1 exhibited an unexpected oncogenic role.	^128^
Pancreatic cancer	Tumor suppressor	- TET1 inhibits the progression and metastasis of pancreatic tumors by impeding the Wnt signaling pathways. It activates SFRP2 transcription by catalyzing demethylation of its promoter, leading to inhibition of both canonical and noncanonical Wnt signaling pathways and impeding epithelial–mesenchymal transition (EMT) in pancreatic tumors.	^63^
Hepatocellular carcinoma (HCC)	Tumor suppressor	- TET1 upregulation in HCC cells inhibits cancer cell growth, migration, invasion, and inflammation while promoting autophagy and apoptosis through the TET1/miR-34a/BACH1/p53 pathway. - Enhanced COX-2 expression in hepatocytes leads to spontaneous HCC formation, associated with DNA promoter hypermethylation driven by reduced TET1 expression.	87, 90
Hepatocellular carcinoma (HCC)	Oncogene	- TET1 upregulation associated with promoting cell proliferation and driving cancer progression in HCC.	^117^
Cholangiocarcinoma (CCA)	Oncogene	- TET1, dependent on 2-oxoglutarate (OG), is significantly upregulated in CCA patients and promotes CCA cell malignancy by increasing 5hmC levels. - Inhibiting TET1 activity suppresses CCA development by influencing cell growth and apoptosis.	^91^

**Figure 4:**
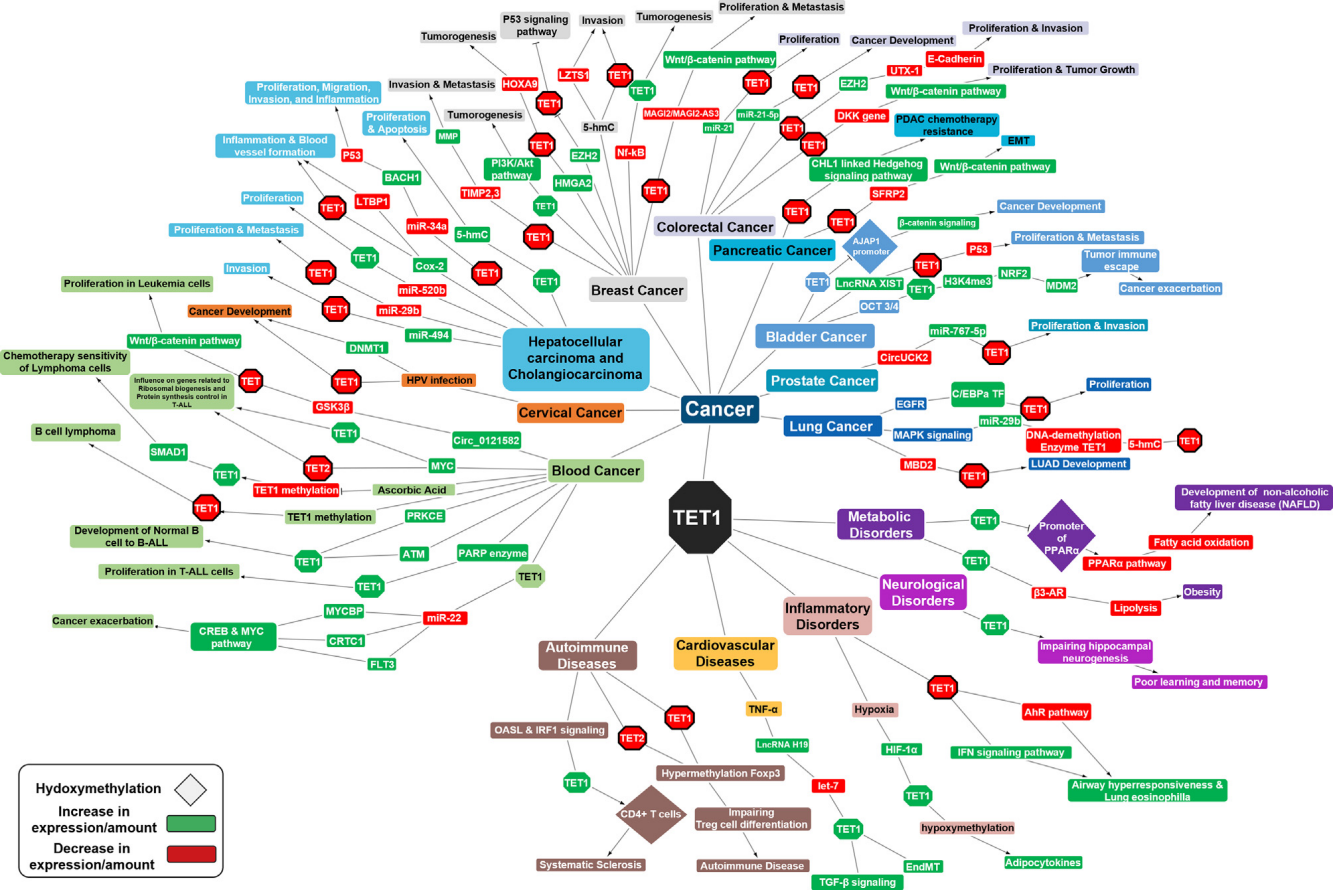
Network visualization of TET1 in clinical disease progression. This figure, created using Cytoscape, provides a comprehensive visualization of TET1's involvement in disease progression. The nodes represent various clinical diseases, while the edges indicate interactions with TET1, illustrating its multifaceted roles across different pathological conditions. Red boxes indicate down-regulation, green boxes represent up-regulation, and triangular shapes signify hydroxymethylation.

Remarkable strides have been made in targeting TET1 for therapeutic purposes, particularly in leukemia, colorectal cancer, triple-negative breast cancer, obesity, and schizophrenia, as summarized in Figure 4. However, the complexity of TET1's multifaceted roles within distinct signaling pathways has presented challenges to its broader clinical applicability. Beyond oncology, TET1 exerts significant influence in diverse areas, including cardiovascular diseases, autoimmune disorders, inflammatory conditions, reproductive and developmental anomalies, metabolic imbalances, and neurological dysfunctions. Yet, these domains require more comprehensive investigation to unlock the full therapeutic potential of TET1. In recent years, new insights have emerged regarding the noncatalytic role of TET1 in early neuronal differentiation, further highlighting its multifaceted nature. The study of TET1 provides a fertile ground for in-depth epigenetic investigations, offering a crucial understanding of the mechanisms underlying various diseases and opening pathways to novel therapeutic targets. The immense potential and substantial value of TET1 in the medical field cannot be overstated. As our knowledge of TET1 deepens, it holds the promise of making invaluable contributions to the field of epigenetics, paving the way for groundbreaking advances in medicine. In this comprehensive review, we have aimed to synthesize a wide array of relevant studies examining the role of TET1 in various clinical conditions. The collective evidence highlights the critical significance of TET1 in the complex landscape of these diseases, providing valuable insights that could guide the development of targeted therapeutic interventions.

## CRediT authorship contribution statement

**Keyvan Jabbari:** Writing – review & editing, Writing – original draft, Visualization. **Ali Khalafizadeh:** Writing – review & editing, Visualization. **Mahboubeh Sheikhbahaei:** Writing – review & editing. **Hossein Soltaninejad:** Writing – review & editing. **Sadegh Babashah:** Writing – review & editing, Supervision, Conceptualization.

## Conflict of interests

The authors declared no conflict of interests.
